# Effects of music therapy in patients with diabetic retinopathy undergoing pan‑retinal photocoagulation

**DOI:** 10.1371/journal.pone.0344435

**Published:** 2026-03-16

**Authors:** Yi Chen, Yiwen Duan, Jiaojiao Zhang, Tingting Cai, Guolan Cao, Changrong Yuan

**Affiliations:** 1 School of Nursing, Fudan University, Shanghai, China; 2 Ophthalmology Outpatient Department, The First Affiliated Hospital of Soochow University, Suzhou, China; Nepal Eye Hospital, NEPAL

## Abstract

**Objectives:**

Pan-retinal photocoagulation is a common outpatient procedure for diabetic retinopathy that often causes procedural pain and anxiety. Nonpharmacologic interventions such as music therapy may provide relief but are underexplored in this population. This study aimed to evaluate the effectiveness of a three-phase music therapy intervention in reducing pain, anxiety, and physiological responses during pan-retinal photocoagulation, and to explore its potential as a supportive outpatient strategy.

**Methods:**

Quasi-experimental study.

**Setting:**

Ophthalmology Outpatient Department, The First Affiliated Hospital of Soochow University.

**Participants:**

Participants were non-randomly allocated. This experimental study allocated participants to groups based on clinic visiting time. Patients attending on Tuesday and Wednesday mornings were assigned to the intervention group, while those attending on Tuesday afternoons formed the control group receiving usual care. This time-based allocation minimized cross-contamination and facilitated implementation in routine practice. 116 patients with diabetes were assigned to experimental or control groups, out of which 59 patients completed the intervention.

**Intervention:**

The intervention consisted of listening to pre-recorded three-phase classical music combined with audiovisual education. The control group only received the audiovisual education.

**Outcome measures:**

Primary outcome was pain, measured by the Visual Analog Scale, it was taken during treatment and 5 minutes after treatment; secondary outcomes included state anxiety (Spielberger State-Trait Anxiety Inventory), heart rate and blood pressure (Omron electronic sphygmomanometer). Theses measurements were taken 15 minutes before and 5 minutes after treatment. Post-treatment satisfaction was evaluated using a study-specific, self-developed questionnaire comprising six items rated on a 5-point Likert scale.

**Results:**

Pain, anxiety, systolic blood pressure and diastolic blood pressure were lower in the intervention group compared with control group (*p* < 0.05). No significant differences in heart rate were observed between groups. There were none adverse events occurred during intervention. Satisfaction was high in both groups, the intervention group rated staff education content and service attitude higher (*p* < 0.05), with no other between-group differences, and music acceptability was favorable.

**Conclusion:**

A three-phase music therapy intervention is a safe and effective approach for reducing procedural pain, anxiety, and blood pressure in patients undergoing PRP. These results support its integration as a nonpharmacologic option in outpatient ophthalmologic care.

**Trial registration number:** ChiCTR2500106330.

## Introduction

Globally, diabetes is a widespread epidemic that affects the health of nearly 537 million people，in 2021, and this number is projected to increase to 784 million by 2045 [1]. The disease imposes a substantial economic burden on patients and carries the risk of numerous disabling and life-threatening complications [2]. One of the most common complications is diabetic retinopathy (DR). A global estimate from 2010 indicated that approximately 4.5 million people suffer from visual impairment or blindness due to this condition [3]. In 2021, an estimated 9.60 million individuals in the United States (26.43% of the diabetic population) were affected by diabetic retinopathy, with 1.84 million (5.06% of diabetic patients) progressing to vision-threatening stages [4,5]. Despite concern about a potential diabetes epidemic in Asia [6], epidemiological data for diabetic retinopathy in Asian countries are scarce. [7] In China, diabetes affects over 90 million people [8], and among them, 15.2 million developed DR [9]. Results of a study [10] in rural China showed that diabetic retinopathy is common, with rates of 43% for any retinopathy and 6.3% for vision-threatening retinopathy. The number of patients suffering from diabetes and related complications is continuously rising in China [11]. DR represents a significant socioeconomic burden on healthcare systems, with its prevalence continuing to rise in aging societies.

Pan-retinal photocoagulation (PRP) is an effective method for preventing the progression of DR and reducing the risk of vision loss [12]. The Diabetic Retinopathy Study recommended that the treatment method was to use a scattering method, using an argon laser with a size of 500 micrometers to cauterize the peripheral retina 800–1600 times [13]. During PRP treatment, laser-induced thermal effects damage retinal tissues at the coagulation sites, triggering the release of prostaglandin-like mediators. This process often causes symptoms such as pain, ocular stinging, pressure, and even headaches.

Pain is a major adverse effect of PRP treatment. As many as 64.1% of patients report being unable to tolerate the pain associated with laser therapy. [14] Evidence summarized in Azarcon’s review suggests that pain is a commonly reported adverse outcome of PRP in advanced diabetic retinopathy [15]. In addition, pain may increase the patient’s risk for complications during the procedure if it stimulates sudden movement of the eye [16]. Pain can hinder patient cooperation during the procedure and may lead some patients to refuse further treatment. Experience of pain may affect compliance to therapy and result to deterioration of vision.

Previous studies have indicated that pain during PRP can be controlled by adjusting laser parameters, including wavelength, duration, and energy flux [17–19]. In addition to laser parameter modulation [20–23], several pharmacological interventions have been used, such as oral nonsteroidal anti-inflammatory drugs, topical diclofenac, inhaled nitrous oxide, oral diazepam, and intramuscular injection of ketorolac tromethamine [24].

Music therapy, as a non-pharmacological intervention, offers advantages over other alternatives such as aromatherapy, hypnosis, and acupuncture. It is simple to administer, non-invasive, cost-effective, and free from adverse effects [25,26]. Music therapy has been widely adopted across healthcare settings and has demonstrated efficacy in reducing both anxiety and pain. Its beneficial effects have been observed in various clinical scenarios, including perioperative care [27], intensive care units [28], labor pain management [29], cancer treatment [30], endoscopic procedures [31–35], invasive punctures [36,37], tube removal [38], and invasive biopsies [39].

However, despite its proven efficacy in many medical contexts, high-quality evidence supporting the use of music therapy to relieve acute procedural pain specifically during PRP remains limited [40]. The American Society for Pain Management Nursing advocates for the right of individuals undergoing potentially painful procedures to receive optimal pain management before, during, and after treatment—including PRP. It further recommends that nurses establish proactive plans to address both pain and anxiety prior to the initiation of such procedures [41].

Moreover, patients’ knowledge and understanding of surgical procedures have been shown to be associated with their anxiety levels. However, individuals undergoing PRP often experience varying degrees of visual impairment, which limits their ability to comprehend treatment-related information presented in standard printed educational materials [42–44]. Studies have demonstrated that audiovisual education is more effective in such contexts [45]. High-quality patient education materials are critical for improving outcomes in patients with DR, as they enhance comprehension and support informed decision-making [45]. Audiovisual education is widely used in ophthalmology and has proven to be an effective method of patient instruction [46].

Therefore, this study will adopt quasi-experimental study to evaluate the effects of a three-phase music intervention, combined with audiovisual education, administered before and during the PRP treatment. The goal is to provide empirical data and theoretical support for the clinical application and implementation of this approach in patients with DR during PRP treatment.

## Methods

### Study design

This was a quasi-experimental non-randomized design and was conducted in an Ophthalmology Clinic in a tertiary comprehensive hospital in Jiangsu Province, China.

Due to the nature of the intervention, blinding was not possible for data collectors and participants; however, the statistical analysts were blinded to group allocation. This study followed the TREND Statement Checklist [47]. This protocol has been reviewed and approved by the Ethics Committee of Soochow University. Approval No. SUDA20231231H02. (Approval date December 31, 2023.) Data collection period was January 10^th^, 2024 (first participant enrolled) to May 2^th^, 2024 (last participant enrolled). The research was conducted in accordance with the principles of the Declaration of Helsinki. Written and verbal informed consent was obtained from all participants. Neither patients nor the public were involved in the design, conduct, or reporting of the study.

### Deviation from the study protocol

According to the original study protocol, participants were to be randomly assigned to the intervention or control group using a list of computer-generated random numbers created in Excel, with group allocations sealed in sequentially numbered opaque envelopes. However, during the actual implementation, this random-number procedure was not applied due to logistical constraints in the outpatient setting and the need to avoid cross-contamination between participants.

Instead, a time-based allocation approach was adopted. Patients attending the ophthalmology clinic on Tuesday and Wednesday mornings were assigned to the intervention group, while those attending on Tuesday afternoons formed the control group receiving usual care. This modification allowed the intervention to be implemented efficiently within the routine clinical workflow while maintaining separation between groups. The change in allocation method was approved by the research team and reported transparently in the present manuscript.

### Participants

The eligibility criteria were the following:

The inclusion criteria: participants were eligible for inclusion if they met the diagnostic criteria for type 2 diabetes and stage III or IV DR. All participants had clear refractive media and were undergoing PRP treatment for the first time.

The exclusion criteria: presence of ocular fundus diseases other than DR, including a history of uveitis, branch retinal vein occlusion, central retinal vein occlusion, macular degeneration, high myopia, or glaucoma; history of ocular laser treatment or eye surgery; recent use of analgesic medications; concurrent mental illness or a family history of psychiatric disorders; severe intellectual or cognitive impairment; severe heart failure or respiratory failure; refusal or inability to tolerate music therapy; pregnancy or lactation.

Elimination criteria: patients who were hospitalized during the study due to other unexpected illnesses or surgeries.

#### Recruitment.

The participants were recruited at The First affiliated Hospital of Soochow University, a tertiary medical facility in Jiangsu Province, China. The participants were recruited by referral from ophthalmologist during the period between January 10th, 2024 to May 2th, 2024. Potential participants were invited to an introductory session, where they were explained about the details of the study and submitted written informed consent.

### Assessments

Assessments were conducted at two time points, including pre-treatment and post-treatment timepoints. Pre-treatment timepoints refer to 15 minutes prior to treatment. Post-treatment timepoints refer to 5 minutes after treatment.

### Laser treatment equipment and procedure

All laser treatments were performed by two fixed ophthalmologists using a standardized laser system: the treatment equipment was Multispectral Ophthalmic Laser Treatment System (Carl Zeiss Visulas Trion), the treatment pulse duration was 200 microseconds, the power was 100 mw to 200 mw. We individualized and titrated the laser energy during the procedure according to each patient’s tolerance and clinical response, consistent with routine clinical practice. In clinical operation, the energy level may vary across individual laser spots; however, due to practical constraints, it was not feasible to record, in real time, the spot-by-spot energy settings and the corresponding pain responses for each patient. Therefore, we reported only the overall energy range used during treatment. Prior to laser application, a topical anesthetic (Oxybuprocaine Hydrochloride Eye Drops) was administered to the ocular surface. The laser procedure commenced immediately after instillation, without a pre-application interval. Notably, the anesthetic was not used for analgesia but rather to facilitate the placement of the procedural contact lens, as per standard operating protocol.

#### Control group.

Audiovisual education, including the face-to-face instruction by the *PowerPoint Presentation* developed by a full-time ophthalmology outpatient nurse, combined with audiovisual materials played by MP3. Power Point and audiovisual equipment were used to deliver preoperative instruction on PRP. The session lasted approximately 5 minutes.

#### Experimental group.

In addition to receiving the same care as the control group, participants in the experimental group were provided with music therapy designed under the guidance of a Music Education Specialist. The playlist was developed based on Music Education Specialist’s professional expertise and clinical experience, with consideration of the local patient population’s general preferences and the treatment context. The intervention was delivered in a standardized, structured manner across predefined phases of the clinical procedure. Details of the playback settings and selected music implemented in the experimental group are in Table 1. As shown in Table 1, the pre-defined playlist was used to ensure intervention consistency across participants, rather than ad hoc selection by the study team.

**Table 1:** Music therapy implemented in the experimental group.

No.	Playback Context	*Music Title*	Duration and Playback Method
1	In a noisy outpatient waiting area, a group of patients waited to be called in for treatment.	*Canon in D Major*	3 minutes 2 seconds per track, looped playback
2	In the waiting area, patients received audiovisual health education. As many were middle-aged or older and unable to fully understand written materials, a video was used to explain the procedure, precautions, cooperation points, and informed consent.	*Always with Me* (*Spirited Away*)	2 minutes 29 seconds per track, played once after video completion
3	While waiting for the patient to enter the treatment room, the doctor prepared by applying three doses of topical anesthetic to the ocular surface.	*Kiss the Rain* *Times Over the Thoughts*	2 minutes 40 seconds 2 minutes 8 seconds Two loops, total approx. 10 minutes
4	The patient was positioned at the slit lamp. The doctor placed the contact lens and activated the laser foot pedal to begin the panretinal photocoagulation procedure.	*Kiss the Rain* *Star River in Your Eyes in C*	2 minutes 40 seconds 3 minutes 47 seconds 1–2 loops, total 5–10 minutes

Music used in the intervention was preloaded onto a USB flash drive and played via a white X4 all-in-one computer (22-inch display, Intel i5 processor, 16 GB RAM, 128 GB storage) connected to a Keling external speaker. The playback volume was maintained at a moderate level (approximately 40–60 dB), ensuring patient audibility without causing discomfort or disrupting clinical communication. Sessions were conducted in a quiet, semi-enclosed environment to minimize external distractions and promote a calm, therapeutic setting.

*Activities to increase compliance* The intervention was embedded in routine outpatient waiting periods, thereby leveraging required wait time to improve adherence. Concurrently, nursing staff offered timely appointment reminders and positive reinforcement to support continued participation.

### Sample size and calculation

The required sample size was calculated using G*Power 3.1.9.4. With a power of 95%, an effect size of 0.38, and a significance level of 0.05, the minimum sample size was determined to be 80 participants (40 per group). To account for potential dropouts or other unforeseen circumstances, the sample size was increased by 28%. Therefore, a minimum of 48 participants per group was targeted, resulting in a total of 116 participants required for this study.

### Allocation method

Participants were non-randomly assigned to groups based on their visiting time. Patients who attended the clinic on Tuesday and Wednesday mornings were allocated to the intervention group, whereas those who attended on Tuesday afternoons were allocated to the control group and received usual care. This time-based allocation was adopted to ensure the feasibility of implementation within the routine outpatient workflow and to minimize contamination between groups. Given the non-random assignment, baseline demographic and clinical characteristics were compared across groups. To standardize implementation and reduce procedural variability, all procedures were conducted in the same clinic room by the same ophthalmologist and the same nurse, following an identical workflow and standardized assessment time points.

### Measurement tools

#### Primary outcome.

The primary outcome was pain. Pain was assessed using the Visual Analogue Scale (VAS). This scale was developed by Price et al. (1983) [48]. Specifically, a 10 cm horizontal line was drawn on paper, with one end marked as 0 (indicating no pain) and the other end marked as 10 (indicating the worst imaginable pain). This scale consists of a 10-cm horizontal or vertical line with the two ends representing the minimum and maximum scores for pain and comfort (0: no pain/no discomfort (very comfort), 10: the most severe pain/very uncomfortable (not comfortable at all) [49]. Pain scores of patients were recorded by making a handwritten mark on a 10-cm line by the researchers.

#### Secondary outcomes.

*State Anxiety Scale.* The State-Trait Anxiety Inventory (STAI), originally developed by Spielberger et al. [50] and later translated into Chinese by Wang Tiansheng and Cheng Zhiping et al. [51], comprises two subscales: State Anxiety (S-AI) and Trait Anxiety (T-AI). The S-AI subscale assesses an individual’s immediate and transient feelings of anxiety, tension, fear, and nervousness, while the T-AI subscale measures more stable and enduring emotional dispositions. In the present study, only the S-AI was used, consisting of 20 items (Items 1–20) that evaluated participants’ current emotional states. Each item was rated on a 4-point Likert scale ranging from 1 (not at all) to 4 (very much so), with total scores ranging from 20 to 80. Higher scores indicated greater levels of state anxiety. Participants were instructed to select the response that best described their feelings at the time of assessment. The test-retest reliability coefficient of the Chinese version of the S-AI was 0.625, with split-half reliability coefficients of 0.781 for males and 0.747 for females.

*Heart Rate*. Heart rate (HR) was assessed as a secondary outcome measure to evaluate the physiological response to the intervention. HR was measured in beats per minute (bpm) using an Omron electronic sphygmomanometer with integrated HR monitoring. Baseline HR was recorded within 15 minutes prior to the PRP procedure, and post-treatment HR was recorded within 5 minutes following the completion of PRP, to evaluate autonomic and emotional responses. HR was treated as a continuous variable in the analysis.

*Systolic and Diastolic Blood Pressure*. Systolic blood pressure (SBP) and diastolic blood pressure (DBP), measured in mmHg, were included as secondary outcome measures to assess physiological responses to the intervention. Blood pressure (BP) was measured using an Omron electronic sphygmomanometer. Baseline SBP and DBP were recorded within 15 minutes prior to the PRP procedure, and post-treatment measurements were obtained within 5 minutes after the completion of PRP.

*Safety assessment*. The study calculated the adverse events as the safety assessment. The researchers recorded the total cases such as hypoglycemic episodes, syncope, arrhythmia and other events, to assess the safety of the music therapy intervention.

*Satisfaction assessment.* Satisfaction was assessed using a study-specific, self-developed questionnaire consisting of six items rated on a 5-point Likert scale (1 = very dissatisfied, 2 = dissatisfied, 3 = neutral, 4 = satisfied, 5 = very satisfied). The intervention group completed all six items, whereas the control group completed items 1, 2, 4, 5, and 6 (item 3, which assessed satisfaction with the type of music used, was applicable only to the intervention group). The items covered satisfaction with (1) the timing of the education session, (2) the format of health education delivery, (3) the type of music used in the program, (4) the educational content provided by staff, (5) staff service attitude, and (6) the overall care process (pre-treatment education–treatment–post-treatment).

### Data collection procedures

All data were collected by a single trained ophthalmology outpatient nurse to ensure consistency. The audiovisual educational intervention was delivered by nurses who had undergone standardized training and followed a unified script and protocol. Data were recorded objectively using pre-designed data collection forms.

Baseline data(pre-treatment) included demographic characteristics, glycated hemoglobin (HbA1c) levels, state anxiety scores measured 15 minutes prior to treatment, and physiological indicators (HR and BP) assessed at the same time point.

Post-treatment data were collected 5 minutes after treatment and included state anxiety scores, HR, BP, pain scores, adverse events occurring during the intervention, and satisfaction ratings.

All data were collected face-to-face in the outpatient clinic setting.

### Statistical analysis

The individual patient was the unit of analysis for assessing intervention effects. Data were entered and verified using Microsoft Excel. All statistical analyses were performed with SPSS version 27.0, with a two-tailed *p*-value < .05 considered statistically significant. Prior to analysis, normality and homogeneity of variance were assessed for all continuous variables. Normality was assessed using the Shapiro–Wilk test and by visual inspection of histograms and Q–Q plots. Homogeneity of variance was evaluated using Levene’s test.

For descriptive statistics, normally distributed continuous variables were expressed as mean ± standard deviation, while non-normally distributed data were presented as median and interquartile range (IQR). Categorical variables were summarized using frequencies and percentages.

For inferential analysis, if the data did not meet assumptions of normality or homogeneity of variance, non-parametric tests such as the Mann–Whitney U test were applied. For normally distributed data, independent sample t-tests and one-way analysis of variance (ANOVA) were used as appropriate.

To explore whether pain during PRP differed by retinal region and whether the intervention effect varied across regions, we fitted a general linear model (univariate) with facial expression pain rating during treatment as the dependent variable and Group (intervention vs control), Region3 (posterior pole, mid-periphery, peripheral region), and the Group × Region3 interaction as fixed factors. Model results are reported as F statistics with degrees of freedom, p values, and partial eta-squared (ηp^2^).

Given the ordinal nature of the pain scale and potential non-normality, we additionally conducted a non-parametric sensitivity analysis comparing Facial2 across Region3 using the Kruskal–Wallis test. Post hoc pairwise comparisons (Dunn’s test with Bonferroni adjustment) were planned only if the omnibus Kruskal–Wallis test was statistically significant.

## Results

### Participants and baseline measurements

**Fig 1:**
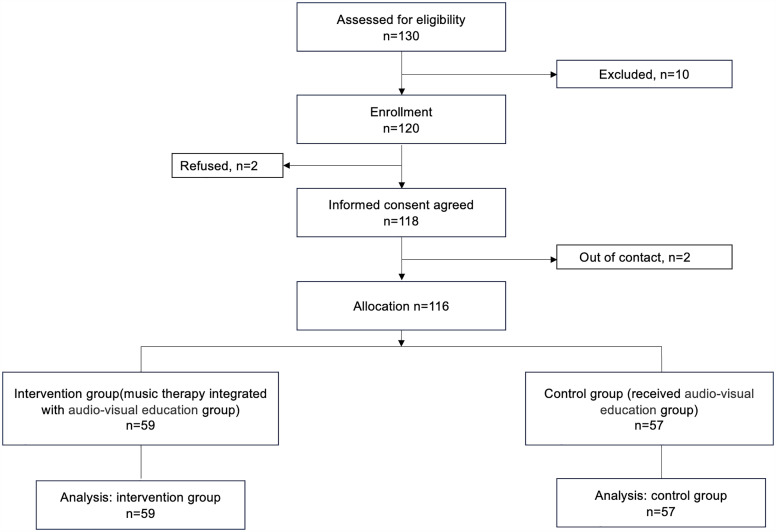
Consolidated Standards of Reporting Trials study flow diagram.

Of the 130 potential participants, ten patients were excluded. During the enrollment, two patients refused, resulting in 118 participants who submitted written informed consent. Two patients were out of contact. Finally, 116 were included in the study. There were 59 patients in the intervention group, while 57 participants in the control group (Fig 1).

A total of 64.7% of participants (75/116) were female. The participants’ average age was 56.34 years old, ranging from 27 to 77. The mean HbA1c was 8.6%, the mean duration of diabetes was 13.57 years, ranging from 0.08 to 32. Participants reported an average duration of 0.94 years (range: 0.02–6 years) since being diagnosed with diabetic retinopathy. The average treatment duration of PRP was 11.35 minutes. The demographic characteristics of the participants were manifested by groups without statistic differences. (Table 2).

**Table 2:** Demographic characteristics of the participants.

Characteristics	Intervention(n = 59) $\overset{―}{x}$ ± s or M (IQR) or n (%)	Control(n = 57) $\overset{―}{x}$ ± s or M (IQR) or n (%)	t/U/F	*p*
Age(year)	55.44 ± 11.44	59(54.36, 60.20)	−0.99	.324
Height	1.66 ± 0.07	1.68(1.63, 1.68)	0.10	.921
Weight	65.41 ± 9.47	65.61 ± 9.24	0.01	.938
BMI	23.62(22.25, 24.51)	23.86 ± 2.48	−0.17	.868
Duration of diabetes	11.96 ± 7.56	15.24 ± 8.76	2.10	.150
HbA1c (%)	8.58(8.17, 9.10)	8.557(8.16, 8.80)	−0.18	.855
How long have you been diagnosed with diabetic retinopathy	0.50(0.57, 1.17)	0.25(0.62, 1.41)	0.04	.967
Treatment duration of PRP	11.00(10.69, 11.58)	11.00(11.05, 12.11)	−1.42	.155
Gender				
Male	38(64.4)	37(64.9)	0.01	.955
Female	21(35.6)	20(35.1)		
Education level				
Primary school or lower	13(22.0)	12(21.1)	0.25	.621
Junior high	14(23.7)	14(24.6)		
Senior high	15(25.4)	19(33.3)		
Associate degree	10(17.0)	8(14.0)		
Bachelor’s degree or above	7(11.9)	4(7.0)		
Marital status				
Married	53(89.8)	50(87.7)	0.92	.341
Single	6(10.2)	4(7.0)		
Divorced or widowed	0(0.0)	3(5.3)		
Employment Status				
Employed	28(47.5)	21(36.8)	0.75	.389
Unemployed	1(1.7)	4(7.0)		
Retired	24(40.7)	25(43.9)		
Farmer	6(10.2)	7(12.3)		
Total monthly household income				
<5999	8(13. 6)	8(14.0)	0.09	.762
6000 ～ 7999	22(37.3)	23(40.4)		
>8000	29(49.2)	26(45.6)		
Treatment site per session				
Left eye	29(49.2)	37(64.9)	2.96	.088
Right eye	30(50.9)	20(35.1)		
Lesion region				
Left posterior pole	10(17.0)	9(15.8)	2.39	.125
Left mid-periphery	8(13. 6)	17(29.8)		
Left peripheral region	11(18.6)	12(21.1)		
Right posterior pole	11(18.6)	8(14.0)		
Right mid-periphery	15(25.4)	7(12.3)		
Right peripheral region	4(6.8)	4(7.0)		

BMI, body mass index; PRP, pan‑retinal photocoagulation

### Intervention adherence and adverse events

During the intervention period, all participants successfully completed the entire music therapy and audiovisual education program without any dropouts or interruptions. No adverse events (such as hypoglycemic episodes, syncope, arrhythmia, or other complications) were reported. Notably, two patients requested to increase the volume to better hear the music.

### Primary outcome

There were no statistically significant differences between the intervention and control groups in pain scores during treatment or in the change in pain scores from baseline (*p* = .351 and *p* = .104, respectively), suggesting comparable pain experiences and levels of pain relief during the procedure. However, post-treatment pain scores differed significantly between the groups (*p* = .031), with the intervention group reporting lower pain levels than the control group. These findings suggest a potential analgesic benefit of music therapy combined with audiovisual education following PRP. (Table 3.)

**Table 3:** Comparison of parameters at the measurement times.

Measurements	Intervention(n = 59)	Control(n = 57)	t/U	*p*
Pain score during treatment	4.17 ± 1.13	4.35 ± 1.52	−0.93	.351
Pain score after treatment	1.69 ± 0.90 ^#^	2.18 ± 1.27 ^#^	−2.15	.031
Change in pain score	−2.47 ± 1.01	−2.18 ± 1.15	−1.63	.104
Pre-treatment state anxiety score	35.44 ± 4.36	38.16 ± 4.98 ^#^	−2.93	.003
Post-treatment state anxiety score	35.49 ± 3.47	39.70 ± 4.15 ^#^	−5.46	<.001
Change in state anxiety score	1(−1.06, 1.16)	1.54 ± 5.11	−0.99	.324
Pre-treatment heart rate	85.29 ± 11.21	85.95 ± 12.95	−0.29	.770
Post-treatment heart rate	82(80.34, 86.10)	83.82 ± 11.87	−0.24	.810
Change in heart rate	−2(−3.67, −0.47)	−3(−3.72, −0.53)	0.10	.923
Pre-treatment systolic blood pressure	136.34 ± 15.86	138.67 ± 18.25	−0.73	.464
Post-treatment systolic blood pressure	132.51 ± 15.71 ^#^	143.23 ± 18.36	−3.51	<.001
Change in systolic blood pressure	−3.83 ± 11.31 ^#^	4.56 ± 31.84 ^#^	−3.34	<.001
Pre-treatment diastolic blood pressure	85.92 ± 9.44	89(84.35, 89.58)	−0.74	.457
Post-treatment diastolic blood pressure	84.88 ± 9.20 ^#^	89.25 ± 8.86	−2.92	.003
Change in diastolic blood pressure	−1.03 ± 5.27	2.28 ± 6.75	−2.96	.004

Annotation: # Despite the non-normal distribution of the data, the mean ± standard deviation was presented to provide a more intuitive comparison of differences between groups.

### Secondary outcome

State anxiety score. Differences in the two groups in pre-treatment state anxiety score and the post-treatment state anxiety score were statistically significant (*p* = .003, *p* < .001, respectively). However, there was no change in state anxiety score after intervention (*p* = .324). (Table 3.)

Heart rate. No significant differences were found between the two groups in pre-treatment, post-treatment, or change in HR (*p* = .770, *p* = .810, *p* = .923, respectively). (Table 3.)

Systolic blood pressure. Significant differences were found between the two groups in both post-treatment SBP and the change from baseline (*p* < .001 for both). In the intervention group, SBP decreased by an average of 3.83 mmHg after treatment, whereas in the control group, it increased by 4.56 mmHg. (Table 3.)

Diastolic blood pressure. Significant differences were observed between the two groups in both post-treatment DBP and the change from baseline (*p* = .003 and *p* = .004, respectively). In the intervention group, DBP decreased by an average of 1.03 mmHg, whereas in the control group, it increased by 2.28 mmHg following treatment. (Table 3.)

### Satisfaction assessment

Satisfaction with the intervention or the education process was assessed both in the intervention and control group in this study (Table 4). Overall, satisfaction ratings were generally high in both groups (medians 4–5 on a 5-point scale). Compared with the control group, the intervention group reported significantly higher satisfaction with the educational content provided by staff (median [IQR]: 5 [5,5] vs 5 [4,5]; Mann–Whitney U = 2191.0, Z = 3.601, p < 0.001) and with the staff’s service attitude (5 [5,5] vs 5 [5,5]; U = 1829.0, Z = 2.316, p = 0.021). No statistically significant between-group differences were observed for satisfaction with the timing of the education session (5 [4,5] vs 5 [4,5]; U = 1980.5, Z = 1.921, p = 0.055), the delivery format of health education (5 [4,5] vs 4 [4,5]; U = 1849.5, Z = 1.056, p = 0.291), or overall satisfaction with the full process (5 [4,5] vs 4 [4,5]; U = 1931.5, Z = 1.597, p = 0.110). Satisfaction with the type of music was assessed only in the intervention group, with a median [IQR] of 4 [4,5] (n = 59), suggesting generally favorable acceptability of the selected music intervention.

**Table 4:** Satisfaction Assessment.

Measurements	Total	Intervention (n = 59)	Control(n = 57)	U	*Z*	*p*
1. How satisfied are you with the timing of this education session?	5(4,5) (n = 116)	5(4,5)	5(4,5)	1980.5	1.921	.055
2. How satisfied are you with the format in which the health education was delivered?	5(4,5) (n = 116)	5(4,5)	4(4,5)	1849.5	1.056	.291
3. How satisfied are you with the type of music used in the program?	4(4,5)(n = 59)	4(4,5)	–	–	–	–
4. How satisfied are you with the educational content provided by the staff?	5(4,5)(n = 116)	5(5,5)	5(4,5)	2191.0	3.601	<.001
5. How satisfied are you with the staff’s service attitude?	5(5,5)(n = 116)	5(5,5)	5(5,5)	1829.0	2.316	.021
6.Overall, how satisfied are you with the entire process (pre-treatment education–treatment–post-treatment)?	4(4,5)(n = 116)	5(4,5)	4(4,5)	1931.5	1.597	.110

### Exploratory findings

For intra-procedural pain assessed using the facial expression pain rating scale, a two-way general linear model with Group (intervention vs control) and retinal region (Region3: posterior pole, mid-periphery, peripheral region) showed no significant Group × Region interaction (F = 0.086, df = 2, *p* = .918; partial η² = 0.002), indicating no evidence that the intervention effect differed by region. The main effect of Group was not significant (F = 0.333, df = 1, *p* = .565; partial η² = 0.003), and the main effect of Region3 was also not significant (F = 1.915, df = 2, *p* = .152; partial η² = 0.034).(Table 5)

**Table 5:** Tests of between-subjects effects for facial expression pain rating during treatment.

Effects	F	df	*P*	Partial eta squared
Group × Region 3	0.086	2	.918	0.002
Main effect of Group	0.333	1	.565	0.003
Main effect of Region3	1.915	2	.152	0.034

Notes: The dependent variable was facial expression pain rating during treatment.

Group: 1=intervention, 0=control.

Region3 was coded as 1 = posterior pole, 2 = mid-periphery,3 =peripheral region.

Results are from a univariate general linear model including Group, Region3, and the Group × Region3 interaction.

Estimated marginal means (EMMs) are presented in Table 6. In the control group, EMMs were 4.118 (SE 0.324; 95% CI 3.476–4.759) for the posterior pole, 4.667 (SE 0.273; 95% CI 4.127–5.207) for the mid-periphery, and 4.125 (SE 0.334; 95% CI 3.464–4.786) for the peripheral region. In the intervention group, corresponding EMMs were 3.905 (SE 0.291; 95% CI 3.327–4.482), 4.435 (SE 0.278; 95% CI 3.883–4.986), and 4.133 (SE 0.345; 95% CI 3.450–4.816), respectively, with substantial overlap of confidence intervals across groups and regions (Table 6).

**Table 6:** Estimated marginal means of facial expression pain ratings during PRP, by group and retinal region.

Group	Region3	Mean	SE	95% CI (Lower–Upper)
0	1	4.118	.324	3.476-4.759
	2	4.667	.273	4.127-5.207
	3	4.125	.334	3.464-4.786
1	1	3.905	.291	3.327-4.482
	2	4.435	.278	3.883-4.986
	3	4.133	.345	3.450-4.816

Note: Values are estimated marginal means from the univariate GLM.

Dependent variable: Facial expression pain rating during treatment.

Group: 0 = control, 1 = intervention.

Region3: 1 = posterior pole, 2 = mid-periphery, 3 = peripheral region.

Additionally, pain scores during treatment did not differ across the three retinal regions (Kruskal–Wallis H = 2.884, df = 2, p = 0.236); therefore, no post hoc pairwise comparisons were conducted.

## Discussion

This study demonstrates that the intervention of music therapy effectively alleviates procedural pain and anxiety in patients with diabetic retinopathy undergoing pan-retinal photocoagulation. The relief achieved in the intervention group was superior to that of patients who received standard audiovisual education without music therapy.

Music is an inexpensive, simple, nonpharmacological method without any side effects, and noninvasive, and has long been utilized as a complementary therapeutic approach [52]. Music influences the right hemisphere of the brain and generates psychophysiological responses through the limbic system. Physiologically, it promotes the release of enkephalins and endorphins, thereby reducing the intensity and perception of pain. Music can also modulate brainwave activity, coordinate muscle tone and movement, and exert an anxiolytic effect [53]. Numerous studies have shown that music therapy effectively reduces pain and anxiety during procedural interventions such as colonoscopy [54], labor [29], prostate biopsy [39], surgery [55], critical care [56], and cancer treatment [57]. For instance, a study conducted in Turkey applied a 30-minute intervention using the Ajam Ashiran maqam of Turkish classical music delivered through MP3 players and headphones before colonoscopy. The results showed that the intervention group reported significantly lower pain and anxiety scores and higher comfort levels compared to the control group (*p* < .050). Following the colonoscopy, patients in the intervention group experienced notable reductions in pain and anxiety, along with increased comfort levels [54]. Findings of the present study are consistent with previous research, further supporting the efficacy of music therapy in alleviating pain and anxiety during procedural interventions. It is worth noting that PRP-related pain is typically most prominent during active laser application, whereas post-procedural discomfort is generally milder. In this study, pain was assessed using a facial expression pain rating scale at two prespecified time points: during PRP, and 5 minutes after PRP. The observation of a between-group difference at 5 minutes post-PRP, but not during the procedure, may be explained by several clinical and methodological considerations. Although PRP-related pain is most intense during active laser delivery, intra-procedural pain is highly variable and may be influenced by spot-by-spot titration of laser parameters and treatment location. Therefore, a single pain rating “during PRP” may have limited sensitivity to detect between-group differences. In contrast, pain assessed 5 minutes post-PRP may better reflect early recovery (e.g., residual discomfort and autonomic arousal) under a more stable environment in which patients can more fully engage in relaxation. Accordingly, the observed reduction in pain at 5 minutes post-PRP may represent improved immediate post-procedural recovery rather than attenuation of peak nociceptive stimuli during laser application.

Moreover, music preference is highly individualized and that patient-selected, preference-based music—particularly when implemented under the guidance of a certified music therapist—may enhance the therapeutic effects of music interventions [27]. In our study, we used a standardized, pre-defined playlist to ensure consistency of exposure across participants and to minimize variability attributable to different musical selections. The playlist was curated by a trained professional with consideration of the local clinical context and general patient preferences; however, it was not individually tailored to each participant’s specific music preference. Future studies should consider allowing participants to select preferred music (or choose from a structured library) and/or incorporating certified music therapists to evaluate whether personalization yields additional benefits in the PRP setting.

This study found that music therapy significantly reduced SBP (by 3.83 mmHg, *p* < .001) and DBP (by 1.03 mmHg, *p* = .030) within 5 minutes after PRP in patients with diabetic retinopathy. In contrast, patients in the control group exhibited increases in both systolic and diastolic blood pressure. No statistically significant difference was observed between the groups in HR changes, which weakens physiological stress arguments. A systematic review and meta-analysis by He et al. reported that music therapy following prostate biopsy only slightly reduced HR, with no significant effect on BP or respiratory rate [39]. This meta-analysis included 24 randomized controlled trials with a total of 1,576 patients. In contrast, another meta-analysis demonstrated that music therapy significantly reduced anxiety, pain, HR, and SBP in patients undergoing cardiothoracic surgery, and even improved oxygen saturation, though no significant effect on DBP was reported [58]. Similarly, in the context of postoperative care after total thyroidectomy, music therapy was shown to accelerate pain relief and produce a greater reduction in respiratory rate compared to standard care [59]. The findings of our study align with existing literature, demonstrating that music therapy can effectively alleviate pain and anxiety, and lower SBP during PRP treatment in patients with diabetic retinopathy. Importantly, our study also found a significant reduction in DBP, which was not observed in prior research. In this study, the observed SBP/DBP changes may reflect modest differences in immediate post-procedural hemodynamic recovery; however, within a short assessment window, HR may be less sensitive to the intervention and/or more influenced by inter-individual variability. Future studies should incorporate more comprehensive stress-related measures (e.g., repeated HR and BP recordings, heart rate variability, and/or biochemical markers) to better characterize autonomic responses. However, as respiratory rate and oxygen saturation were not monitored in this study, conclusions regarding the effects of music therapy on these physiological parameters could not be drawn.

With the increasing integration of digital technologies in healthcare, personalized approaches to music therapy are becoming more feasible. For example, recent studies have explored the use of digital platforms to assess participants’ musical preferences and emotional states in real time, allowing for tailored music selection that better meets individual needs [57]. Future interventions could adopt similar precision-based strategies to optimize music therapy protocols. This would not only expand the range of clinically applicable music interventions but also enhance the quality of care for patients experiencing procedural pain and anxiety in outpatient settings.

This study has several limitations. First, the nature of the intervention precluded blinding of outcome assessors, which may have introduced observer bias. Second, as the study was conducted at a single center, the external validity and generalizability of the findings to other clinical settings may be restricted. Third, the music intervention was implemented during routine outpatient waiting time with nursing support, which likely facilitated adherence; however, this delivery context may not be readily replicable in settings without structured waiting periods or dedicated personnel. Fourth, individual- and region-specific differences in music preferences may limit the generalizability of our findings. Fifth, participants were allocated by clinic time blocks and no adjustment analyses were performed; therefore, residual confounding due to unmeasured factors cannot be excluded, including time-related influences (e.g., circadian variation in anxiety/mood), commuting-related stress, glycemic fluctuations, clinic crowding, and other time-varying clinic conditions. Additionally, consistent with evidence from a systematic review [24], laser parameters (e.g., energy/power and exposure time) may affect PRP-related pain. Because detailed, spot-by-spot laser settings were not recorded in real time, we could only report the overall parameter range, which may limit interpretation of pain outcomes. Future studies should adopt randomized allocation and/or counterbalanced scheduling (e.g., delivering both conditions across multiple time blocks) and consider prespecified adjustment strategies to further mitigate bias.

## Conclusions

This study evaluated the effects of a three-phase music intervention administered before and during PRP in patients with diabetic retinopathy. The intervention effectively reduced patients’ pain and anxiety levels and led to decreases in both systolic and diastolic blood pressure in the intervention group. These findings provide empirical evidence and theoretical support for the clinical application of this combined approach in outpatient PRP settings. Based on our results, music therapy appears to be a safe and effective strategy for alleviating procedural pain and anxiety in patients undergoing PRP.

### Strengths and limitations of this study

1. This study provides evidence for an effective, low-cost, and nonpharmacologic intervention to manage procedural pain and anxiety in outpatient ophthalmologic care.
2. The integration of structured music therapy into routine nursing education protocols offers a replicable model for enhancing patient comfort and physiological stability during invasive procedures.
3. The external validity of our findings could be constrained by the single-center design.

## Supporting information

S1 File Protocol. (DOCX)

S2 File Multimedia Files. Music.
(JPG)

S3 File TREND Statement Checklist. (PDF)

S4 File Survey Questionnaire. (DOCX)

S5 File Dataset. (XLSX)

S6 File Dataset Variable Coding Scheme. (DOCX)

## Abbreviations

DR: diabetic retinopathy
PRP: pan-retinal photocoagulation
TREND: transparent reporting of evaluations with nonrandomized designs
VAS: visual analogue scale
STAI: the state-trait anxiety inventory
S-AI: state anxiety
T-AI: trait anxiety
HR: heart rate
Bpm: beats per minute
SBP: systolic blood pressure
DBP: diastolic blood pressure
BP: blood pressure
HbA1c: glycated hemoglobin
IQR: interquartile range
ANOVA: one-way analysis of variance.
