# Supplementary material for: Effects of music therapy in patients with diabetic retinopathy undergoing pan‑retinal photocoagulation
Source: PLoS One. 2026 Mar 16;21(3):e0344435. doi: 10.1371/journal.pone.0344435 (PMC12991246; doi:10.1371/journal.pone.0344435)
Supplement: S1 File — (DOCX) [file pone.0344435.s001.docx]

Protocol

| Diabetes mellitus has become a global epidemic, affecting nearly 500 million people worldwide. This disease imposes a significant economic burden on patients and carries the risk of multiple disabling and life-threatening complications, among which diabetic retinopathy (DR) is one of the most prevalent. Global estimates (2010) indicate that approximately 4.5 million people suffer from vision impairment or blindness due to DR. By diabetes type, DR affects 42.1% of patients with type 1 diabetes and 25.5% of those with type 2 diabetes. DR represents a substantial socioeconomic cost to healthcare systems, and its prevalence continues to rise in aging societies.  Panretinal photocoagulation (PRP) is an effective intervention to halt DR progression and reduce the risk of vision loss. During PRP treatment, laser-induced thermal effects damage the photocoagulated retinal tissue, triggering the release of prostaglandin-like mediators that can cause ocular stinging, distending pain, and even headaches. These painful symptoms often lead to poor patient compliance during treatment and may result in refusal of subsequent sessions.  Studies have shown that music therapy can alleviate both anxiety and pain in patients. Additionally, since outpatient DR patients often have visual impairments that make text-based health education unsuitable, audiovisual education has been demonstrated to be more effective. Therefore, this study aims to implement a combined intervention of music relaxation therapy and audiovisual education prior to PRP treatment, evaluating its effects on anxiety levels, pain intensity, adverse reactions (e.g., syncope), and patient satisfaction in DR patients.  1. Research Methods  (1) Study Design: Randomized controlled trial  (2) Study Participants  Using convenience sampling, eligible DR patients will be recruited from the ophthalmology clinics of tertiary hospitals between December 2023 and December 2024.  The eligibility criteria were the following：  The inclusion criteria: participants were eligible for inclusion if they met the diagnostic criteria for type 2 diabetes and stage III or IV DR. All participants had clear refractive media and were undergoing PRP treatment for the first time.  The exclusion criteria: presence of ocular fundus diseases other than DR, including a history of uveitis, branch retinal vein occlusion, central retinal vein occlusion, macular degeneration, high myopia, or glaucoma; history of ocular laser treatment or eye surgery; recent use of analgesic medications; concurrent mental illness or a family history of psychiatric disorders; severe intellectual or cognitive impairment; severe heart failure or respiratory failure; refusal or inability to tolerate music therapy; pregnancy or lactation.  Elimination criteria: patients who were hospitalized during the study due to other unexpected illnesses or surgeries.  3) Sample Size and Calculation Method  (1) Sample Size and Calculation  The required sample size was calculated using G*Power 3.1.9.4. With a power of 95%, an effect size of 0.38, and a significance level of 0.05, the minimum sample size was determined to be 80 participants (40 per group). To account for potential dropouts or other unforeseen circumstances, the sample size was increased by 28%. Therefore, a minimum of 48 participants per group was targeted, resulting in a total of 116 participants required for this study.  (2) Randomization method  Random grouping was carried out by the researchers using the list of computer random numbers generated by Excel. A random number table was referenced, starting from row 1, column 2. Odd numbers were assigned to Group A (intervention group), and even numbers to Group B (control group), until 48 participants were enrolled in each group. The random numbers and group assignments were stored together in sealed envelopes. After eligible participants provided informed consent, the researcher G.L. C opened the envelope and recorded the assigned group.  4) Measurement Tools  Primary Outcome. The primary outcome was pain. Pain was assessed using the Visual Analogue Scale (VAS). This scale was developed by Price et al. (1983) [48]. Specifically, a 10 cm horizontal line was drawn on paper, with one end marked as 0 (indicating no pain) and the other end marked as 10 (indicating the worst imaginable pain). This scale consists of a 10-cm horizontal or vertical line with the two ends representing the minimum and maximum scores for pain and comfort (0: no pain/no discomfort (very comfort), 10: the most severe pain/very uncomfortable (not comfortable at all) [49]. Pain scores of patients were recorded by making a handwritten mark on a 10-cm line by the researchers.  Secondary Outcomes  State Anxiety Scale. The State-Trait Anxiety Inventory (STAI), originally developed by Spielberger et al. [50] and later translated into Chinese by Wang Tiansheng and Cheng Zhiping et al. [51], comprises two subscales: State Anxiety (S-AI) and Trait Anxiety (T-AI). The S-AI subscale assesses an individual’s immediate and transient feelings of anxiety, tension, fear, and nervousness, while the T-AI subscale measures more stable and enduring emotional dispositions. In the present study, only the S-AI was used, consisting of 20 items (Items 1–20) that evaluated participants’ current emotional states. Each item was rated on a 4-point Likert scale ranging from 1 (not at all) to 4 (very much so), with total scores ranging from 20 to 80. Higher scores indicated greater levels of state anxiety. Participants were instructed to select the response that best described their feelings at the time of assessment. The test-retest reliability coefficient of the Chinese version of the S-AI was 0.625, with split-half reliability coefficients of 0.781 for males and 0.747 for females.  Heart Rate. Heart rate (HR) was assessed as a secondary outcome measure to evaluate the physiological response to the intervention. HR was measured in beats per minute (bpm) using an Omron electronic sphygmomanometer with integrated HR monitoring. Baseline HR was recorded within 15 minutes prior to the PRP procedure, and post-treatment HR was recorded within 5 minutes following the completion of PRP, to evaluate autonomic and emotional responses. HR was treated as a continuous variable in the analysis.  Systolic and Diastolic Blood Pressure. Systolic blood pressure (SBP) and diastolic blood pressure (DBP), measured in mmHg, were included as secondary outcome measures to assess physiological responses to the intervention. Blood pressure (BP) was measured using an Omron electronic sphygmomanometer. Baseline SBP and DBP were recorded within 15 minutes prior to the PRP procedure, and post-treatment measurements were obtained within 5 minutes after the completion of PRP.  Safety assessment. The study calculated the adverse events as the safety assessment. The researchers recorded the total cases such as hypoglycemic episodes, syncope, arrhythmia and other events, to assess the safety of the music therapy intervention.  Data Collection Procedures  All data were collected by a single trained ophthalmology outpatient nurse to ensure consistency. The audiovisual educational intervention was delivered by nurses who had undergone standardized training and followed a unified script and protocol. Data were recorded objectively using pre-designed data collection forms.  Baseline data(pre-treatment) included demographic characteristics, glycated hemoglobin (HbA1c) levels, state anxiety scores measured 15 minutes prior to treatment, and physiological indicators (HR and BP) assessed at the same time point.  Post-treatment data were collected 5 minutes after treatment and included state anxiety scores, HR, BP, pain scores, adverse events occurring during the intervention, and satisfaction ratings.  All data were collected face-to-face in the outpatient clinic setting.  （5）Intervention  Control Group.  Audiovisual education, including the face-to-face instruction by the PowerPoint Presentation developed by a full-time ophthalmology outpatient nurse, combined with audiovisual materials played by MP3. Power Point and audiovisual equipment were used to deliver preoperative instruction on PRP. The session lasted approximately 5 minutes.  Experimental Group.  In addition to receiving the same care as the control group, participants in the experimental group were provided with music therapy designed under the guidance of a Music Education Specialist. The music-based relaxation intervention was administered in a structured manner during different phases of the clinical process. Details of the playback settings and selected music are in Table 1.  Table 1. Music therapy implemented in the experimental group.   \| No. \| Playback Context \| Music Title \| Duration and Playback Method \| \| --- \| --- \| --- \| --- \| \| 1 \| In a noisy outpatient waiting area, a group of patients waited to be called in for treatment. \| Canon in D Major \| 3 minutes 2 seconds per track, looped playback \| \| 2 \| In the waiting area, patients received audiovisual health education. As many were middle-aged or older and unable to fully understand written materials, a video was used to explain the procedure, precautions, cooperation points, and informed consent. \| Always with Me (Spirited Away) \| 2 minutes 29 seconds per track, played once after video completion \| \| 3 \| While waiting for the patient to enter the treatment room, the doctor prepared by applying three doses of topical anesthetic to the ocular surface. \| Kiss the Rain  Times Over the Thoughts \| 2 minutes 40 seconds  2 minutes 8 seconds  Two loops, total approx. 10 minutes \| \|  \|  \|  \|  \| \| 4 \| The patient was positioned at the slit lamp. The doctor placed the contact lens and activated the laser foot pedal to begin the panretinal photocoagulation procedure. \| Kiss the Rain  Star River in Your Eyes in C \| 2 minutes 40 seconds  3 minutes 47 seconds  1–2 loops, total 5–10 minutes \|   （6）Data Collection Procedures  All data were collected by a single trained ophthalmology outpatient nurse to ensure consistency. The audiovisual educational intervention was delivered by nurses who had undergone standardized training and followed a unified script and protocol. Data were recorded objectively using pre-designed data collection forms.  Baseline data(pre-treatment) included demographic characteristics, glycated hemoglobin (HbA1c) levels, state anxiety scores measured 15 minutes prior to treatment, and physiological indicators (HR and BP) assessed at the same time point.  Post-treatment data were collected 5 minutes after treatment and included state anxiety scores, HR, BP, pain scores, adverse events occurring during the intervention, and satisfaction ratings.  All data were collected face-to-face in the outpatient clinic setting.  2．Statistical Analysis  Data were entered and verified using Microsoft Excel. All statistical analyses were performed with SPSS version 27.0, with a two-tailed p-value < 0.05 considered statistically significant. Prior to analysis, normality and homogeneity of variance were assessed for all continuous variables.  For descriptive statistics, normally distributed continuous variables were expressed as mean ± standard deviation, while non-normally distributed data were presented as median and interquartile range (IQR). Categorical variables were summarized using frequencies and percentages.  For inferential analysis, if the data did not meet assumptions of normality or homogeneity of variance, non-parametric tests such as the Mann–Whitney U test were applied. For normally distributed data, independent sample t-tests and one-way analysis of variance (ANOVA) were used as appropriate.  3. Technical Roadmap  **Trial**  **Enrollment**  **Enrollment**  **Baseline Data Collection：**demographic characteristics, glycated hemoglobin (HbA1c)、state anxiety score (assessed 15 minutes pre-treatment)、physiological parameters (measured 15 minutes pre-treatment) :heart rate、blood pressure  **Experimental Group（n=59）**  **Control Group (n=57)**  **Music therapy+** **Audiovisual Education**  **Audiovisual Education**  **Outcome Evaluation**  State Anxiety Score at 5 minutes post-treatment  Heart rate and blood pressure at 5 minutes post-treatment  Pain Score at 5 minutes post-treatment  Adverse events during treatment  **Analysis and Conclusion**  Date：December 18^th^, 2023 |
| --- | --- | --- | --- | --- | --- | --- | --- | --- | --- | --- | --- | --- | --- | --- | --- | --- | --- | --- | --- | --- | --- | --- | --- | --- |
