# Supplementary figures and images for: Effects of music therapy in patients with diabetic retinopathy undergoing pan‑retinal photocoagulation
Source: PLoS One. 2026 Mar 16;21(3):e0344435. doi: 10.1371/journal.pone.0344435 (PMC12991246; doi:10.1371/journal.pone.0344435)

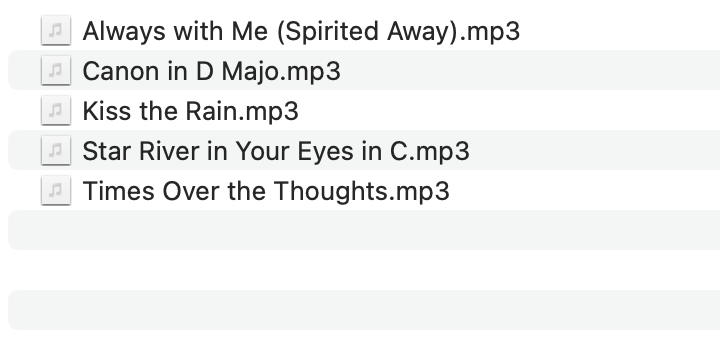

Supplement: S2 File — Music. (JPG) [file pone.0344435.s002.jpg]
