# Supplementary material for: Effects of music therapy in patients with diabetic retinopathy undergoing pan‑retinal photocoagulation
Source: PLoS One. 2026 Mar 16;21(3):e0344435. doi: 10.1371/journal.pone.0344435 (PMC12991246; doi:10.1371/journal.pone.0344435)
Supplement: S4 File — (DOCX) [file pone.0344435.s004.docx]

**Survey Questionnaire on Treatment-Related Aspects of Panretinal Photocoagulation (PRP)**

**I. Demographic Information Questionnaire**
Outpatient ID No.: ________ Date: ____ / ____ / ____ (YYYY/MM/DD)

Please select the option that best describes your situation by placing a “√” on the corresponding number, or fill in the blank “___” truthfully. Unless otherwise indicated, each item is single choice.

1. Date of birth: ____ / ____ / ____ (YYYY/MM/DD)
2. Sex: ① Male ② Female
3. Educational level: ① Primary school or below ② Junior high school ③ Senior high school ④ College/Associate degree ⑤ Bachelor’s degree or above
4. Marital status: ① Married ② Unmarried ③ Divorced ④ Widowed
5. Current employment status: ① Employed ② Unemployed ③ Retired
6. Total household monthly income:
   ① < CNY 2,000/month
   ② CNY 2,000–3,999/month
   ③ CNY 4,000–5,999/month
   ④ CNY 6,000–7,999/month
   ⑤ ≥ CNY 8,000/month
7. Ethnicity: ① Han ② Hui ③ Manchu ④ Uyghur ⑤ Other (please specify): ________
8. Duration of diabetes: ____ years
9. HbA1c level in the past 3 months: ____ %
10. How long have you been aware of retinopathy? (enter a number)
11. Lesion laterality: ① Left eye ② Right eye ③ Both eyes
    Treatment eye this time: ① Left eye ② Right eye ③ Both eyes

Area (severity) of diagnosed lesions—please mark “X” (assuming the patient is facing the investigator):


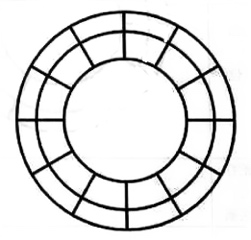

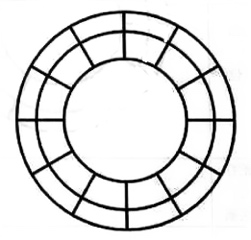


Left Right

12. Do you have any comorbidities? Please mark “√” for all that apply:

① Myocardial infarction

② Congestive heart failure

③ Peripheral vascular disease

④ Cerebrovascular disease or TIA

⑤ Hemiplegia

⑥ Dementia/Alzheimer’s disease

⑦ Chronic obstructive pulmonary disease (COPD)/asthma

⑧ Rheumatoid arthritis or connective tissue disease

⑨ Peptic ulcer disease

⑩ Diabetes mellitus

⑪ Moderate to severe chronic kidney disease

⑫ Liver disease

⑬ Solid tumor

⑭ Leukemia

⑮ Lymphoma

⑯ HIV/AIDS

Other: __________

13. Other measures

Height: ____ cm Weight: ____ kg

15 min before treatment: Heart rate ____ Blood pressure ____

5 min after treatment: Heart rate ____ Blood pressure ____

**II. State–Trait Anxiety Inventory (STAI)**
(To be completed 15 minutes before treatment:)

| **Item** | **Statement** | Not at all | Somewhat | Moderately | Very much |
| --- | --- | --- | --- | --- | --- |
| 1 | I feel calm. |  |  |  |  |
| 2 | I feel secure. |  |  |  |  |
| 3 | I am tense. |  |  |  |  |
| 4 | I feel strained. |  |  |  |  |
| 5 | I feel at ease. |  |  |  |  |
| 6 | I feel upset. |  |  |  |  |
| 7 | I am presently worrying over possible misfortunes. |  |  |  |  |
| 8 | I feel satisfied. |  |  |  |  |
| 9 | I feel frightened. |  |  |  |  |
| 10 | I feel comfortable. |  |  |  |  |
| 11 | I feel self-confident. |  |  |  |  |
| 12 | I feel nervous. |  |  |  |  |
| 13 | I am jittery. |  |  |  |  |
| 14 | I feel indecisive. |  |  |  |  |
| 15 | I feel relaxed. |  |  |  |  |
| 16 | I feel content. |  |  |  |  |
| 17 | I am worried. |  |  |  |  |
| 18 | I feel confused. |  |  |  |  |
| 19 | I feel steady. |  |  |  |  |
| 20 | I feel pleasant. |  |  |  |  |

(To be completed 5 minutes after treatment:)

| **Item** | **Statement** | Not at all | Somewhat | Moderately | Very much |
| --- | --- | --- | --- | --- | --- |
| 1 | I feel calm. |  |  |  |  |
| 2 | I feel secure. |  |  |  |  |
| 3 | I am tense. |  |  |  |  |
| 4 | I feel strained. |  |  |  |  |
| 5 | I feel at ease. |  |  |  |  |
| 6 | I feel upset. |  |  |  |  |
| 7 | I am presently worrying over possible misfortunes. |  |  |  |  |
| 8 | I feel satisfied. |  |  |  |  |
| 9 | I feel frightened. |  |  |  |  |
| 10 | I feel comfortable. |  |  |  |  |
| 11 | I feel self-confident. |  |  |  |  |
| 12 | I feel nervous. |  |  |  |  |
| 13 | I am jittery. |  |  |  |  |
| 14 | I feel indecisive. |  |  |  |  |
| 15 | I feel relaxed. |  |  |  |  |
| 16 | I feel content. |  |  |  |  |
| 17 | I am worried. |  |  |  |  |
| 18 | I feel confused. |  |  |  |  |
| 19 | I feel steady. |  |  |  |  |
| 20 | I feel pleasant. |  |  |  |  |

**III. Facial Expression Pain Rating Scale**

- **15 minutes before treatment: Please choose one facial expression that best reflects your current level of pain and mark “√” on the line.**

**
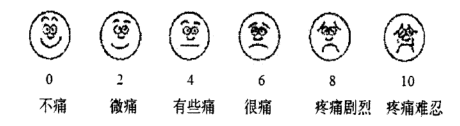
**

No pain Mild Moderate Severe Very severe Unbearable pain

- **During treatment: Please choose one facial expression that best reflects your current level of pain and mark “√” on the line.**

**
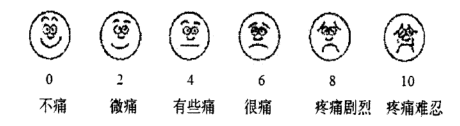
**

No pain Mild Moderate Severe Very severe Unbearable pain

- **5 minutes after treatment: Please choose one facial expression that best reflects your current level of pain and mark “√” on the line.**


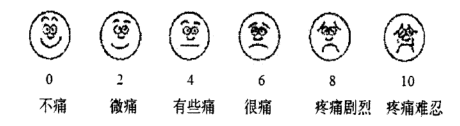


No pain Mild Moderate Severe Very severe Unbearable pain

**IV. Satisfaction Questionnaire**

Response options: 1 = Very dissatisfied, 2 = Dissatisfied, 3 = Neutral, 4 = Satisfied, 5 = Very satisfied.

1. How satisfied are you with the timing of this education session?
   A. Very satisfied B. Satisfied C. Neutral D. Dissatisfied E. Very dissatisfied
2. How satisfied are you with the format in which the health education was delivered?
   A. Very satisfied B. Satisfied C. Neutral D. Dissatisfied E. Very dissatisfied
3. How satisfied are you with the type of music used in the program?
   A. Very satisfied B. Satisfied C. Neutral D. Dissatisfied E. Very dissatisfied
4. How satisfied are you with the educational content provided by the staff?
   A. Very satisfied B. Satisfied C. Neutral D. Dissatisfied E. Very dissatisfied
5. How satisfied are you with the staff’s service attitude?
   A. Very satisfied B. Satisfied C. Neutral D. Dissatisfied E. Very dissatisfied
6. Overall, how satisfied are you with the entire process (pre-treatment education–treatment–post-treatment)?
   A. Very satisfied B. Satisfied C. Neutral D. Dissatisfied E. Very dissatisfied

**V. Adverse Event Record During Treatment**

Treatment start time: ________
Treatment duration: ________
Treatment end time: ________

| **Item** | **Please check** |
| --- | --- |
| Hypoglycemic reaction | ☐ Yes ☐ No |
| Syncope | ☐ Yes ☐ No |
| Arrhythmia | ☐ Yes ☐ No |
| Other | ☐ Yes ☐ No |
