# Supplementary material for: Effects of music therapy in patients with diabetic retinopathy undergoing pan‑retinal photocoagulation
Source: PLoS One. 2026 Mar 16;21(3):e0344435. doi: 10.1371/journal.pone.0344435 (PMC12991246; doi:10.1371/journal.pone.0344435)
Supplement: S6 File — (DOCX) [file pone.0344435.s006.docx]

**Variable Coding Scheme**

| **Variable** | **Coding** |
| --- | --- |
| Group | 1=intervention,0=control |
| Age | continuous variable |
| Q4Sex | 1 = male; 2 = female |
| Q5Educationlevel | 1 = Primary school or below;  2 = Junior high school;  3 = Senior high school;  4 = College/Associate degree;  5 = Bachelor’s degree or above |
| Q6Maritalstatus | 1 = Married;  2 = Unmarried;  3 = Divorced;  4 = Widowed |
| Q7Employmentstatus | 1 = Employed;  2 = Unemployed;  3 = Retired |
| Q8Householdincome | 1 = <CNY2,000; 2 = CNY 2,000–3,999; 3 = 4,000–5,999; 4 = 6,000–7,999; 5 = ≥8,000 |
| Q9Ethnicity | 1 = Han;  2 = Hui;  3 = Manchu;  4 = Uyghur;  5 = Other |
| Q10Diabetesyears | continuous variable |
| Q11HbA1c | HbA1c in the past 3 months (%);1000=missing values |
| Q12Retinopathyyears | Time since retinopathy was detected (years) |
| Q13Lesionlaterality | 1 = left eye;  2 = right eye;  3 = both eyes |
| Q14Treatmenteye | 1 = left eye;  2 = right eye;  3 = both eyes |
| Q15Diagnosedlesionarea | 1 = left posterior pole; 2 = left mid-periphery; 3 = left peripheral region; 4 = right posterior pole; 5 = right mid-periphery; 6 = right peripheral region |
| Q16Comorbities | 1–16; 17 = Other—please specify disease name)  1=Myocardial infarction;2= Congestive heart failure;3=Peripheral vascular disease;4=Cerebrovascular disease or TIA;5=Hemiplegia;6=Dementia/Alzheimer’s disease;7=COPD/asthma;8= Rheumatic disease or connective tissue disease；9=  Peptic ulcer disease；10=Diabetes mellitus；11=Moderate-to-severe chronic kidney disease；12=Liver disease；13= Solid tumor；14=Leukemia；15=Lymphoma；16=HIV/AIDS |
| Height | continuous variable |
| Weight | continuous variable |
| BMI | continuous variable |
| HR1 | continuous variable，heart rate 15 min before treatment |
| SBP1 | continuous variable, systolic blood pressure 15 min before treatment |
| DBP1 | continuous variable, diastolic blood pressure 5 min after treatment |
| HR2 | heart rate 5 min after treatment |
| SBP2 | systolic blood pressure 5 min after treatment |
| DBP2 | diastolic blood pressure 5 min after treatment |
| Pain1 | facial expression pain rating, during treatment) (0 = No pain; 2 = Mild pain; 4 = Moderate pain; 6 = Severe pain; 8 = Very severe pain; 10 = Unbearable pain |
| Pain2 | facial expression pain rating, 5 min after treatment) (0 = No pain; 2 = Mild pain; 4 = Moderate pain; 6 = Severe pain; 8 = Very severe pain; 10 = Unbearable pain |
| S1satisfactionrating | satisfaction rating, 5 = Very satisfied; 4 = Satisfied; 3 = Neutral; 2 = Dissatisfied; 1 = Very dissatisfied |
| S2satisfactionrating | satisfaction rating, 5 = Very satisfied; 4 = Satisfied; 3 = Neutral; 2 = Dissatisfied; 1 = Very dissatisfied |
| S3satisfactionrating | satisfaction rating, 5 = Very satisfied; 4 = Satisfied; 3 = Neutral; 2 = Dissatisfied; 1 = Very dissatisfied |
| S4satisfactionrating | satisfaction rating, 5 = Very satisfied; 4 = Satisfied; 3 = Neutral; 2 = Dissatisfied; 1 = Very dissatisfied |
| S5satisfactionrating | satisfaction rating, 5 = Very satisfied; 4 = Satisfied; 3 = Neutral; 2 = Dissatisfied; 1 = Very dissatisfied |
| S6satisfactionrating | satisfaction rating, 5 = Very satisfied; 4 = Satisfied; 3 = Neutral; 2 = Dissatisfied; 1 = Very dissatisfied |
| Starttime | Treatment start time |
| Treatmentduration | Treatment duration (min) |
| Endtime | Treatment end time |
| Hypoglycemicreaction | 1 = Yes; 0 = No |
| Syncope | 1 = Yes; 0 = No |
| Arrhythmia | 1 = Yes; 0 = No |
| otherresponse | 1 = Yes; 0 = No |
| BeSTAI | STAI Scores before treatment |
| AfterSTAI | STAI Scores after treatment |
| changeSTAI | Change in STAI score (post-treatment-pre-treatment) |
| Q11HbA1cSMEAN | Q11HbA1cSMEAN is an SPSS-generated variable created by Series Mean imputation. It represents HbA1c values after replacing missing observations (e.g., values defined as missing) with the overall mean HbA1c of the dataset. |
| changeHR | Change in heart rate (post-treatment-pre-treatment) |
| changeSBP | Change in systolic blood pressure (post-treatment-pre-treatment) |
| changeDBP | Change in diastolic blood pressure (post-treatment-pre-treatment) |
| changePain | Change in pain rating (post-treatment-pre-treatment) |
| Region3 | 1 = posterior pole; 2 = mid-periphery; 3 =peripheral region |
